# A synbiotic mixture for the management of infantile colic: A randomized trial

**DOI:** 10.1007/s00431-024-05860-5

**Published:** 2024-11-19

**Authors:** Hanne Delcourt, Koen Huysentruyt, Yvan Vandenplas

**Affiliations:** Vrije Universiteit Brussel (VUB), UZ Brussels, KidZ Health Castle, Laarbeeklaan 101, 1090 Brussels, Belgium

**Keywords:** Infant colic, Probiotic, Synbiotic, Quality of life

## Abstract

Infant colic is defined as a recurrent and prolonged period of fussing, crying and/or irritability that cannot be prevented or resolved by caregivers. The aim of this study is to evaluate the efficacy of a synbiotic (Bactecal D Liquid) in infants consulting a primary health care professional for inconsolable crying. A randomized trial was conducted in 68 infants diagnosed by the consulted primary health care professional as “probably suffering from infant colic”. Patients were randomized into two groups and given the synbiotic once (group 1) or twice (group 2) a day for 28 days. Quality of life (QoL) of the caregivers, evaluated with a Likert scale, was the primary outcome. Secondary outcomes included the total number of crying episodes, total crying time, gassiness and “balling of the fists”. The median (Q1;Q3) QoL scores were significantly (*p* < 0.001) higher on day 28 than at baseline: 6 (5;7) vs 2 (1;3). At baseline, there was no significant difference (*p* = 0.527) in QoL between both groups. The improvement in QoL was already significant after one week of intervention for both groups. The median number of crying episodes, overall crying time, gassiness and “balling of fists” were significantly lower on day 28 compared to baseline (*p* < 0.001).

*Conclusion*: The synbiotic tested was shown to be efficacious in the management of infant colic. A significant improvement was observed after 7 days of intervention, which is much earlier than the expected decrease related to the natural evolution of infant colic.

**Table:** 

What is Known: *• Some probiotic strains are reported to be effective in the management of infants presenting with colic, if breastfed.*
What is New: *• The synbiotic studied improved quality of life of caregivers of infants presenting infant colic.* *• Two doses of the synbiotic were not more effective than one dose.* *• The improved occurred within one week.* *• The improvement was independent of feeding (breastfeeding, formula feeding or mixed feeding).*

What is Known:

*• Some probiotic strains are reported to be effective in the management of infants presenting with colic, if breastfed.*

What is New:

*• The synbiotic studied improved quality of life of caregivers of infants presenting infant colic.*

*• Two doses of the synbiotic were not more effective than one dose.*

*• The improved occurred within one week.*

*• The improvement was independent of feeding (breastfeeding, formula feeding or mixed feeding).*

## Introduction

Historically, there have always been infants who did cry more than others. Infant colic (IC) is a common cause of inconsolable crying. The original definition for IC by Wessel et al. dates from 1954 and is known as “The Rule of Three”: colic is defined as an infant crying during more than 3 h per day, more than 3 days per week, for longer than 3 weeks [1]. However, a precise measurement of the duration of infant crying is almost impossible and is impracticable. Therefore, the Rome IV criteria, dating from 2016, define IC as a recurrent and prolonged period of fussing, crying and/or irritability which cannot be prevented or resolved by caregivers [2].

IC typically starts during the first week of life and peaks around six weeks of life and resolves in almost all infants by the age of five months [3]. The reported prevalence ranges between 10 to 40% of all infants [4]. Despite decades of research, the aetiology of IC remains unknown. Several hypotheses have been proposed, such as increased intestinal permeability, chronic intestinal inflammation and dysbiosis [5, 6]. Dysbiosis might be the key mechanism since correction of the dysbiosis decreases permeability and inflammation[6, 7]. Dysbiosis is characterized by a disruption to the microbiome resulting in an imbalance in the microbiota, changes in their functional composition and metabolic activities, or a shift in their local distribution [6].

Although IC is traditionally considered as a benign, self-limiting process in otherwise healthy infants [4], long term consequences are reported [8, 9]. Most studies display an association between IC and the onset of functional gastrointestinal disorders (FGIDs) years later, probably related to the presence of common etiopathogenetic factors (environmental, dietary, intestinal dysmotility, visceral hypersensitivity) [9]. A relationship between IC and subsequent headaches, of the migraine type are reported [9]. Similarly, behavioural problems in children with a history of IC appear to be associated with higher parental stress scores [9]. The current evidence is based on associations, and a causal relationship between IC and long-term consequences was not documented. Also, it is not known if effective treatment has an impact on these long-term consequences.

An effective management of IC will increase the quality of life (QoL) of the infants and their family [3, 4, 10, 11]. Probiotics (mainly *Limisolactobacillus reuteri* DSM 17938 and *Bifidobacterium animalis lactis* BB-12) have been reported to decrease IC in breastfed infants [10]. The International Scientific Association for Probiotics and Prebiotics (ISAPP) published consensus papers on the definition of pro-, pre- and synbiotics. Probiotics are defined as “live microorganisms that, when administrated in adequate amounts, confer a health benefit on the host” [12]. Their definition of a prebiotic is “a substrate that is selectively utilized by host microorganisms conferring a health benefit” [13]. An ISAPP panel updated the definition of a synbiotic to “a mixture comprising live microorganisms and substrate(s) selectively utilized by host microorganisms that confers a health benefit on the host” [14]. A complementary synbiotic has not been designed so that its component parts function cooperatively must be composed of a probiotic plus a prebiotic. A synergistic synbiotic does not need to be so. A synergistic synbiotic is a synbiotic for which the substrate is designed to be selectively utilized by the co-administered microorganisms [14].

The aim of this study is to evaluate the efficacy of Bactecal D Liquid®, a multistrain synbiotic, in infants consulting a primary health care professional (HCP) for inconsolable crying.

## Methods

A randomized trial was conducted in infants diagnosed by the consulted HCP as “probably suffering from IC”. This study was performed between 8th April 2022 and 16th October 2023.

Full term infants (37–41 weeks amenorrhea) with a birthweight > 2750 g, presumed healthy and between 2 and 8 weeks old, presenting with symptoms suggesting IC as defined by Rome IV criteria [2] were included. All types of feeding were allowed: exclusive breastfeeding, mixed feeding and formula feeding.

Exclusion criteria included: preterm infants, infants with acute or chronic illness as judged by the investigator, the prior intake of probiotics as a treatment, malnutrition (weight-for-height ratio < 5%) and parents unable to understand the requirements of study participation. The administration of other medications or food supplements was not allowed.

All outcomes were assessed at baseline and weekly until the end of the trial, on day 28. A consultation with the HCP was planned at inclusion and at day 28, and was possible whenever the parents felt the need for an extra contact. Changes in QoL of the caregivers was the primary outcome measure of this study. QoL was assessed by a self-score Likert scale, ranging from 1 (worst) to 7 (best), at baseline, day 7, 14 and day 28. Secondary outcomes included the number of crying episodes and total crying time both assessed at three well-defined time points during the day (morning, afternoon, night). Gassiness of the infant and “balling of the fists” were both also assessed by a Likert scale ranging from 1 (worst) to 7 (best).

There were two study groups. Patients were randomized into two groups and given a synbiotic once or twice a day for the duration of 28 days. Randomization was done via a total of 80 identical envelopes indicating that the patient was randomized in Group 1 or Group 2. Each participating HCP received 6 envelopes, but the envelope-number was determined by a computer. The first group received one dose of Bactecal Liquid® (Table 1: composition). The second group received two doses of Bactecal Liquid®. Doses were given at well-defined time points in the morning and the evening.

**Table 1:**
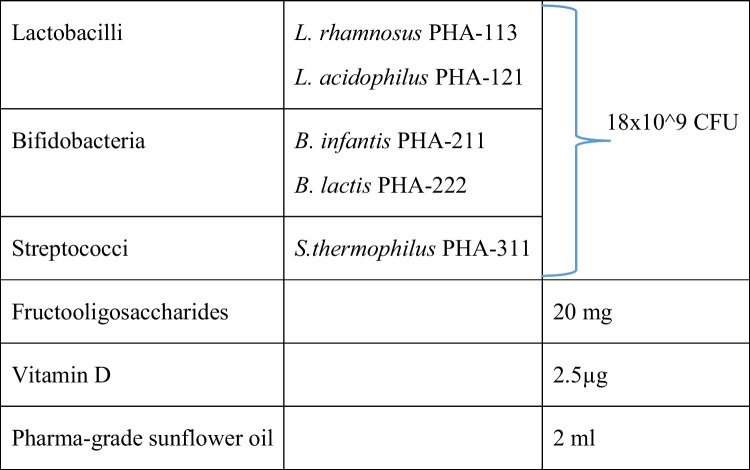
Composition of Bactecal Liquid® per dose

*CFU*, Colony Forming Units; *L*, *Lacticaseibacillus* or *Lactobacillus*; *B*, *Bifidobacterium*; *Str*, *Streptococcus*; *mg*, milligram; *µg*, micrograms; *ml*, milliliter

Descriptive statistics were used to summarize baseline characteristics of all participants. Continuous variables were summarized using means and standard deviations or medians and interquartile ranges, while categorical variables were summarized using frequencies and percentages. The normality of the distribution was checked visually using qq-plots and more formally using a Shapiro–Wilk test. Baseline imbalances were tested using a Wilcoxon rank sum test, χ^2^-test and a Fisher Exact test where appropriate.

The analyses were conducted on an intention-to-treat (ITT) basis, including all participants according to their randomized treatment assignment. For the primary outcome measure, between-group differences at day 28 were assessed using a paired Wilcoxon rank sum test. Linear mixed models were utilized for adjusting for potential confounding variables. Model fit was assessed using diagnostic plots and goodness-of-fit statistics. All statistical analyses were performed using R version 4.1.3. A two-tailed significance level of α = 0.05 was used for all hypothesis tests, and confidence intervals were calculated at the 95% level.

The required sample size was calculated to detect at least 1 unit change in parent QoL score between the baseline and the day 28, with a power of 80% and confidence of 0.05, at a dropout rate of 30%. The sample size needed was 58.

The study was registered (NCT05052476). The Ethical Committee of the UZ Brussel approved the protocol (BUN1432021000622). The research was performed in accordance with the Declaration of Helsinki. Both parents signed the informed consent before starting the study.

## Results

The total study population consists of 68 patients, 37 in group 1 (one dose of the synbiotic) and 31 in group 2 (two doses of the synbiotic). As the recruitment was carried out in parallel at the various centers, the number of inclusions was slightly higher than initially planned (n = 58). The median (Q1;Q3) age was 1.1 (0.8; 1.4) months; 42% of patients were boys. There was no statistically significant difference in baseline characteristics between both intervention groups. The characteristics of all included infants at baseline are listed in Table 2.

**Table 2:** Baseline characteristics

	All patients	Group 1 (1 dose of synbiotic combination)	Group 2 (2 doses of synbiotic combination)	*p*-value
Number	68	37 (54%)	31 (46%)	0.364
Age median (Q1;Q3) (months)	1.1 (0.8;1.4)	1.2 (0.9;1.5)	1.0 (0.7;1.3)	0.177
Sex* (Boys)	42.0%	48.6%	34.5%	0.248
Smoking	30.9%	24.3%	38.7%	0.201
C-section^$^	26.8%	31.2%	20.8%	0.544
Feeding* Breastfeeding Formula feeding Mixed	56.1% 37.9% 6.1%	57.1% 37.1% 5.7%	54.8% 38.7% 6.5%	1.000

*C-section*, Cesarean section; *n = 66; ^$^*n* = 56

**Fig. 1:**
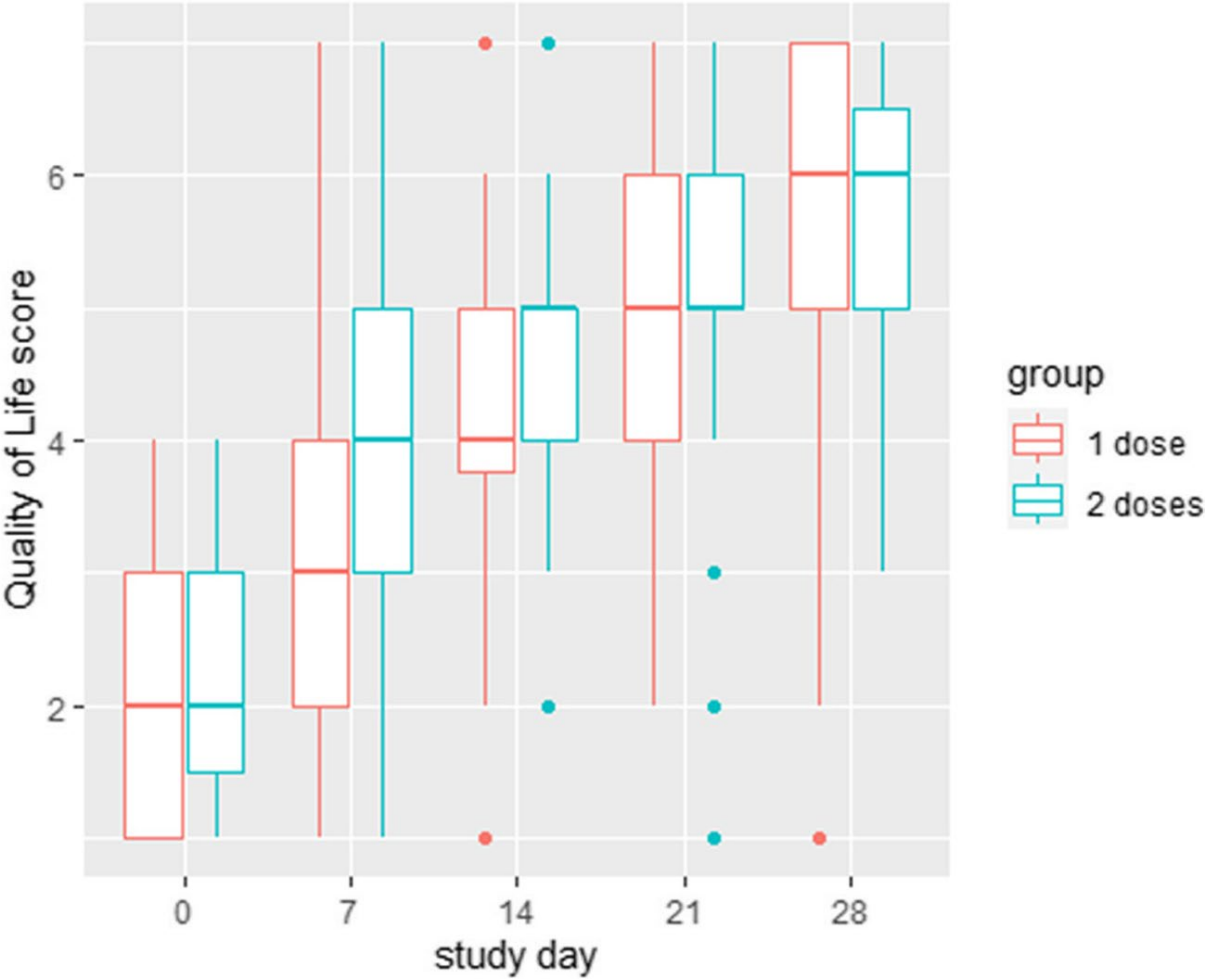
Evolution of quality of life scores during the treatment period

**Fig. 2:**
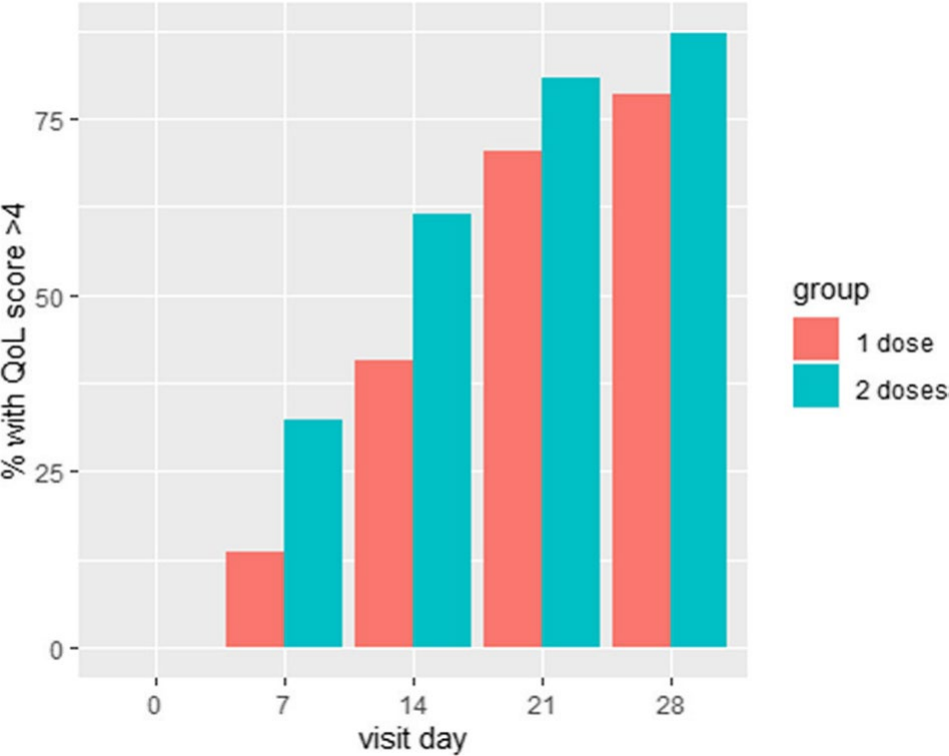
Distribution of QoL score > 4 during treatment period

Regarding the primary outcome, QoL, the overall median (Q1;Q3) scores were significantly (p < 0.001) higher on day 28 than on baseline: 6 (5;7) vs 2 (1;3) (Table 3**; **Fig. 1). There was no significant difference (*p* = 0.924) in QoL between both study groups on day 28: 6 (5;7) vs 6 (5;6.5). The improvement in QoL was significant already at day 7 in both groups (adjusted *p*-value < 0.001 for both groups). However, the difference between both groups was not significant at any time point, even without correcting for multiple testing. The overall median (Q1;Q3) QoL on day 7 was 3.5 (2;4), while it was 3 (2;4) in the 1 dose group and 4 (3;5) in the 2 doses group. A post hoc analysis using a linear mixed model, using breastfeeding as a reference showed no significant association between QoL-improvement and feeding mode (formula feeding 0.26 (95% CI − 0.17;0.70), mixed feeding 0.31 (95% CI − 0.60;1.21)). The distribution of parents with a QoL > 4 was significantly different between day 0 and 28. QoL improved in both groups in the same way. There was no difference in the number of parents reporting QoL above 4 (Fig. 2, Appendix Table 4).

**Table 3:** Quality of life (expressed using Likert scale)

		Group 1	Group 2	*P*
Inclusion				
	Median	2	2	0.527
	Q1–Q3	1–3	1–3	
	Range	1–4	1–4	0.655
Day 28	Mean	5.4	5.6	
	Median	6	6	0.924
	Q1–Q3	5–7	5–6.5	
	Range	1–7	3–7

The median number (Q1;Q3) of crying episodes was significantly (*p* < 0.001) lower on day 28 than at baseline: 8 (5;10) vs 3 (2;4). This was also the case in as well the one-dose (*p* < 0.001) as the two-dose (*p* < 0.001) group. There was no significant difference (*p* = 0.378) in the total number of crying episodes between both study groups on day 28: 3 (2;4.5) vs 3 (2;4) (Appendix Table 5). At baseline, there was no difference in the number of crying episodes in the morning, afternoon or night (Appendix Figure 3).

The median (Q1;Q3) overall crying time was significantly (*p* < 0.001) lower on day 28 than on baseline: 5 (4;6.9) vs 2 (0.8;3) hours. There was no significant difference (*p* = 0.905) in the total crying time between both study groups on day 28: 1.5 (1;3) vs 2 (0.7;3.2) hours (Appendix Table 6). There was no difference in duration of crying during the morning, afternoon or night at baseline (Appendix Figure 4). At no time point, there was a difference in the decrease of duration of crying between both groups (Appendix Figure 5).

There was also no difference in gassiness or “balling of fists” between both groups at baseline (Appendix Figure 6, Appendix Figure 7) any time point, but both parameters also improved significantly over time (data not shown).

## Discussion

The synbiotic tested was shown to be efficacious in the management of IC. Up to now, the primary outcome of the majority of the RCTs on IC was always “duration of crying” and “number of crying episodes” [15, 16]. However, this parameter is subject to be biased as there is no objective measurement of the duration of the crying episodes, and infants may also be crying for other reasons. Therefore, we used “quality of life” as primary outcome, because we considered QoL to be the most relevant parameter for the parents, since colic is considered a benign, self-limiting condition [3]. QoL improved significantly in this trial, and this from the first week onwards, and continued to improve up to day 28, independent of the type of feeding. Duration of crying, number of episodes of crying, gassiness and “balling of the fists” were secondary outcomes which all improved similar in both groups.

We choose for a comparison of the efficacy between two dosages, as there are no dose-efficacy data yet available. Moreover, given the fact that (i) there are many “anti-colic” products available on the market, and (ii) the stress that the unconsolable crying causes to parents, parents refuse the option to give placebo. Parents do not accept the risk to administer a placebo since many products claiming efficacy in infantile colic are available on the market.

We evaluated a synbiotic containing five different strains and a small amount of fructo-oligosaccharides (FOS). Since the product does contain FOS and probiotics, it has to be considered a symbiotic. However, the content of FOS is small (20 mg/dosage), in comparison with the amount of human milk oligosaccharides in mother's milk (~ 10 g/l). Therefore, the therapeutic effect is more likely to be the probiotic than the prebiotic effect. Dysbiosis has been shown to be present in IC, with a decreased presence of bifidobacteria and an increased presence of the gas-producing proteobacteria [5]. Therefore, gassiness was chosen also as secondary outcome. Literature reviews conclude that there is sufficient evidence to recommend *L. reuteri* DSM 17938 *B. lactis* BB-12 in breastfed infants with IC[10].

The absence of a placebo is a limitation of this trial. However, many parents of a baby with IC do refuse placebo because their QoL is low and there are products on the market claiming efficacy. Therefore, some trials are comparative trials between two products, such as simeticone in one group and a probiotic in the other group [17]. According to the systematic review by Wolke, including 8690 infants, the natural evolution of IC is characterized with a peak up to 25% at the age of 5–6 weeks, and decreases to < 5% at 10–12 weeks [18]. According to the Rome IV criteria infantile colic can last up to the age of 5 months [2]. The median age of inclusion in our trial was 5 weeks (1.1 month) and the duration of intervention was 4 weeks. This means that at the end of the trial, the median age of the infants was only 9 weeks or 2.1 months. Moreover, a significant improvement was observed after 7 days of intervention, what is much earlier than the decrease related to the natural evolution. This makes the likelihood that the improvement in QoL was due to the natural evolution of IC small, although it cannot be formally excluded. Even in the case the improvement of all criteria would be related to a placebo effect, QoL of parents (and infants) improved significantly. Smoking and mode of delivery were unrelated to the outcome of the trial. Regarding the impact of mode of feeding, probiotic trials could only show a benefit in breastfed babies [15].

In conclusion: the synbiotic tested was found to improve QoL of parents that have a baby with IC within one week of intervention, independent of the type of feeding. Using breastfeeding as a reference, the QoL-improvement was comparable in the mixed and exclusive formula fed groups.

## Figures and Tables

**Fig. 3:**
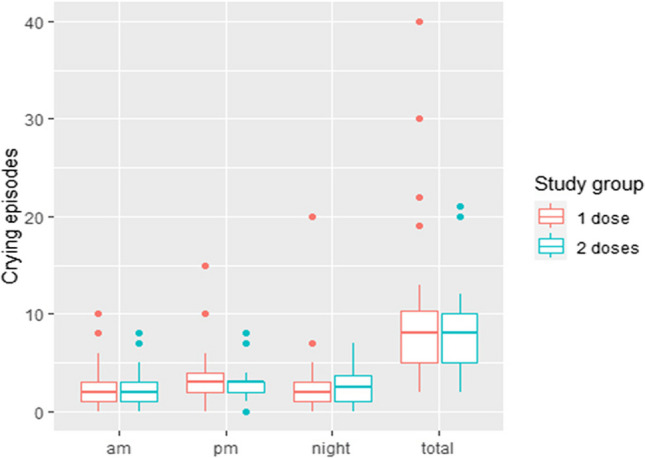
Number of episodes of crying at inclusion. No difference in number of episodes of crying at inclusion (morning, afternoon, night, all day)

**Fig. 4:**
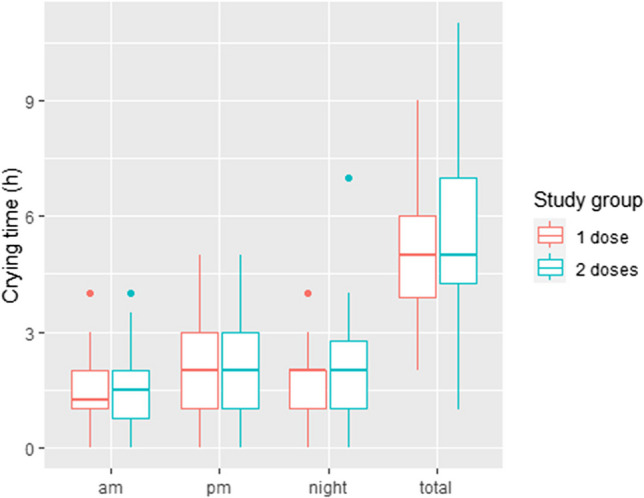
Duration of crying at inclusion. No difference in crying time at inclusion (morning, afternoon, night, all day)

**Fig. 5:**
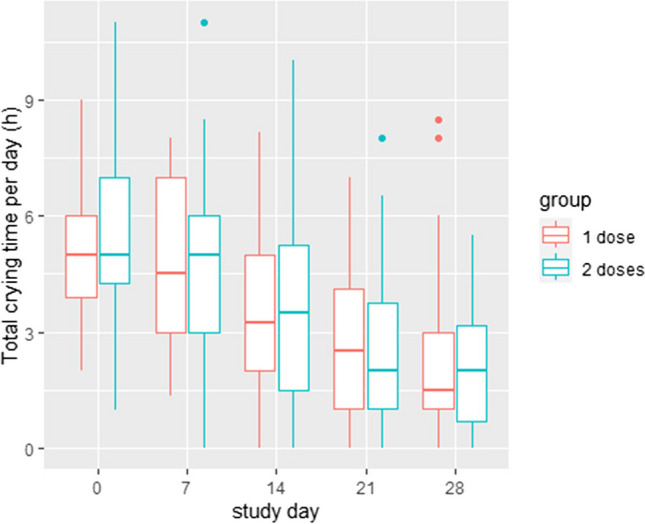
Evolution of crying time (hours). At no time point there was a difference in duration of crying time between both groups

**Fig. 6:**
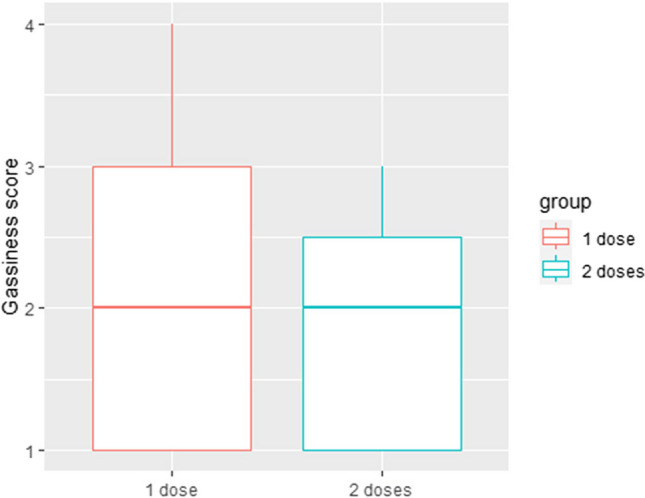
Gassiness at inclusion. No difference in gassiness at inclusion

**Fig. 7:**
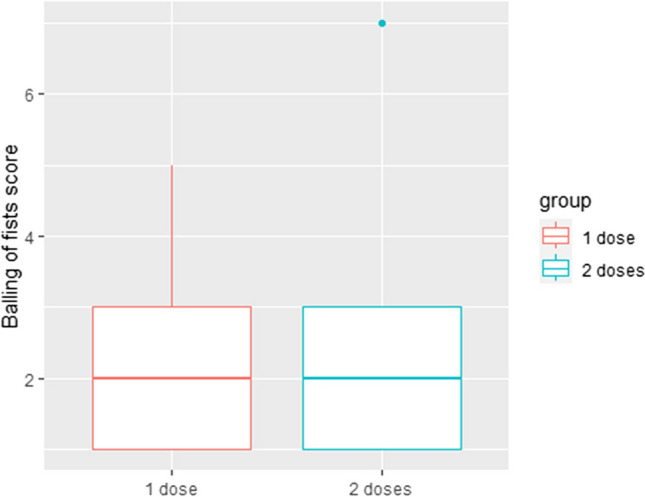
“Balling of fists” at inclusion. No difference in “balling of fists” at inclusion

## Data Availability

No datasets were generated or analysed during the current study.

## Appendix

**Table 4:** Within group numbers and percentages of caregivers with QoL > 4 at each time point

	Group 1 (1 dose)	Group 2 (2 doses)
Inclusion	0%	0%
Day 7	13.5%	32.3%
Day 14	40.5%	61.3%
Day 21	70.3%	80.6%
Day 28	78.4%	87.1%

**Table 5:** Number of episodes of crying /day

		Group 1	Group 2	*p*-value
Inclusion	Mean	9	8	0.712
	Median	8	8	
	Q1–Q3	5–10	5–10	
	Range	2–40	2–21	
Day 28	Mean	4	3	0.378
	Median	3	3	
	Q1–Q3	2–4.5	2–4	
	Range	0–22	0–7	
*p*-value		< 0.001	< 0.001	

**Table 6:** Duration of crying/day (hours)

		Group 1	Group 2	*p*-value
Inclusion	Mean	5.1	5.6	0.363
	Median	5	5	
	Q1–Q3	3.9–6.0	4.2–7.0	
	Range	2–9	1–11	
Day 28	Mean	2.3	2.2	0.905
	Median	1.5	2	
	Q1–Q3	1–3	0.7–3.2	
	Range	0–8.5	0–5.5	
*p*-value		< 0.001	< 0.001	

## Abbreviations

B: Bifidobacteria
FOS: Fructo-oligosaccharides
HCP: Health care professional
IC: Infant colic
ITT: Intention to treat
Lim: *Limisolactobacillus *(formerly called* Lactobacillus*)
RCT: Randomized controlled trial
QoL: Quality of life
